# Survival Percentile and Predictors of Difference in Survival among Hemodialysis Patients and Their Additive Interaction Using Laplace Regression

**DOI:** 10.34172/jrhs.2020.32

**Published:** 2020-11-14

**Authors:** Salman Khazaei, Mehdi Yaseri, Vida Sheikh, Maryam Nazemipour, Ebrahim Hazrati, Mohammad Ali Mansournia

**Affiliations:** ^1^Research Center for Health Sciences, Hamadan University of Medical Sciences, Hamadan, Iran; ^2^Department of Epidemiology and Biostatistics, Tehran University of Medical Sciences, Tehran, Iran; ^3^Clinical Research Development Unit of Shahid Beheshti Hospital, Hamadan University of Medical Sciences, Hamadan, Iran; ^4^Psychosocial Health Research Institute, Iran University of Medical Sciences, Tehran, Iran; ^5^Department of Anesthesiology, AJA University of Medical Sciences, Tehran, Iran

**Keywords:** Hemodialysis, Kidney failure, Survival, Laplace regression

## Abstract

**Background:** Identifying survival modifiable factors and additive interaction between them could help in prioritizing the clinical care of Hemodialysis (HD) patients. We aimed to examine the survival rate and its predictors in HD patients; and explore the additive interaction between survival modifiable factors.

**Study design:** A retrospective cohort study.

**Methods:** The present study was performed on 1142 HD patients in Hamadan Province, western Iran from 2007 to 2017. Data were collected through a researcher-made checklist on hospital records. Laplace regression was used to evaluate differences in 40^th^ survival percentiles in different levels of predictors as well as exploring the pairwise additive interactions between variables.

**Results:** We observed significantly higher survival in nonsmoker patients (40^th^ percentile difference = 5.34 months, 95% CI: 2.06, 8.61). Survival was shorter by more than 3 years in CRP positive patients (40^th^ percentile difference=36.9 months, 95% CI: 32.37, 41.42). Patients with normal albumin (40^th^ percentile difference =24.92, 95% CI: 18.04, 31.80) and hemoglobin (40^th^ percentile difference = 18.65, 95% CI: 12.43, 24.86) had significantly higher survival (*P*<0.001). There was super-additive interaction between being CRP negative and nonsmoker (β_3_ = 9.42 months, 95% CI: 3.35, 15.49 (*P*=0.002)).

**Conclusion:** High CRP and low serum albumin and hemoglobin were associated with the increased risk of death in HD patients. The results of this study support the presence of super-additive interaction between CRP status with serum hemoglobin and also CRP status with smoking, resulting in excess survival in HD patients.

## Introduction

Globally, chronic kidney disease (CKD) is a major health concern and an excessive cost of health care finances devoted to this problem ^1^. The survival rate of these patients is lower than the general population, and it was not seen any improvement in their survival over recent years ^2^.

Hemodialysis (HD) in Iran is generally used as the main choice for renal replacement therapy in end-stage renal disease (ESRD) patients and is provided free of charge in Iran ^3^. Until now, limited studies have been performed in Iran regarding the survival of HD patients, indicating a low survival rate for these patients ^4,5^.

According to litterateurs, age, ethnic background, level of serum albumin and hemoglobin, adequacy of dialysis, mean duration of dialysis per treatment session, renal replacement therapy method, body mass index (BMI), causes of kidney failure, and comorbidities with some diseases such as heart failure and cancer are considered as predictors of death in the HD patients ^3,6-9^.

The interaction generally occurs as a result of the dependence of the influence of one risk factor to the presence of another risk factor. When there is additive interaction between the two risk factors that the patients with both risk factors simultaneously have higher risk of death than those expected based on summing the separate effects of the above-mentioned two risk factors ^10^. Assessing interaction provides a better vision into the mechanisms for the occurrence of outcome and identify more beneficiary subgroups to be intervened when resources are limited. Therefore it is one of the stimulants for assessing additive interaction ^10,11^. A strikingly increase showed in mortality risk among dialysis patients which was due to interaction between protein-energy wasting (PEW), cardiovascular diseases (CVD) and inflammation in patients ^12^.

 Identification of the additive interaction between survival modifiable factors could help in prioritizing the clinical care of HD patients. To our knowledge, there was no previous study done on the additive interaction between survival modifiable factors in hemodialysis patients, moreover, evidence about the survival of hemodialysis patients and its related factors in developing countries is limitedd.

 Therefore, the purposes of the present study were (1) evaluating the survival rate and its predictors in HD patients in Hamadan province, and (2) investigating the additive interaction between the survival modifiable factors in HD patients.

## Methods

###  Study Design

 Retrospective cohort study

###  Settings and Participants

 We examined data obtained from 1142 hemodialysis patients in Hamadan Province, western Iran in the period of 11 years from Mar 2007 to Mar 2017. Hamadan Province is located in the west of Iran and has an area of 19,493 square km in extent and 1,758,268 population according to the national census by the Statistical Center of Iran in 2011. Information was obtained from the eight hospitals of the province with dialysis centers including Alimoradian, Besat, Vali-asr, Ghaem, Imam Hossein, Valiasr, Shahid-Beheshti and Imam Reza in Nahavand, Hamadan, Tuyserkan, Asadabad, Malayer, Razan, Hamadan and Kabudarahang city, respectively.

 Patients undergoing HD due to acute renal failure, patients undergoing peritoneal dialysis, patients on transient hemodialysis and patients who had incomplete medical records were not included in the study and were considered as exclusion criteria.

###  Clinical and Demographic Measures

 Data were collected using a checklist on hospital records of all HD patients hospitalized in provincial hospitals. The checklist used in this study included characteristics related to demographic profiles (age, gender, marriage status, Body mass index (BMI), location, educational level, previous history of smoking or substance abuse), and patient biochemical and clinical information (Hemoglobin level, C-reactive protein (CRP) status (+/-), blood urea nitrogen (BUN), creatinine, urea reduction ratio (URR), sodium, phosphor, calcium, albumin and etiology of ESRD). Clinical and biochemical data at the time of diagnosis and before onset of the first dialysis were gathered for each patient as well and considered as baseline data. To minimize measurement variability, we averaged both baseline measures for each patient. These records were gathered by assessing patients’ medical records in the dialysis ward.

###  Outcomes

 We considered death due to renal failure as the endpoint of the study. Patients with renal transplantation, withdrawal of dialysis, lost-to-follow-up, competing risks (patients who died due injury, accident, or other causes unrelated to renal failure) and those transferred to another dialysis facility out of province were treated as censored cases.

###  Analytical Methods

Laplace regression was used to evaluate differences in survival percentiles according to the levels of predictors and adjusting for potential confounders. Laplace regression as a flexible method can be used for computing the conditional percentiles of the time-to-event variables ^13^. The time that the specific percentage of the investigated cases have experienced the outcome of interest can be considered as survival percentile for that time.

To avoid extrapolation, we examined the 40^th^ percentile of survival time given that during the study period, death occurred for 43% of participants. Therefore, using Laplace regression, we estimated differences in the time duration by which the first 40% of the HD patients died according to the levels of predictors. We also assessed pairwise, additive interactions between predictors.

To assess the effect of the two binary predictors (e.g. G and E) and their additive interaction on the 40^th^ survival percentile (*p*_(40)_), we fitted the following Laplace model:

 "T(p(40)│G,E)=β_0 (p(40))+β_1 (p(40))G+β_2 (p(40))E+β_3 (p(40)).G.E"

According to the above equation the measure of additive interaction between two predictors is the parameter β_3_(p_(40)_). If β_3_(p_(40)_) >0, superadditive interaction between two predictors exists and If β_3_(p_(40)_) <0, interaction is subadditive ^14^.

 We analyzed the data using Stata software version 12 (Stata Corp LP, College Station, Texas) at less than 5% significant level.

###  Ethical approval

 The Ethics Committee of Tehran University of Medical Sciences (TUMS.SPH.REC.1395.1300) approved our study. To keep confidentiality, all patients identifier were removed.

## Results

The baseline characteristics of HD patients are shown in Table 1. Of the 1142 HD patients, 617 (54.03%) were male, 717 (62.78%) were urban resident and 928 (81.26%) of them were married. The mean age (SD) at diagnosis and the mean BMI (SD) of patients was 55.52 (14.65) year and 23.26 (3.54) kg/m^2^, respectively. Among all the subjects; 256 (22.42%) were smokers and 385 (34.65%) were CRP positive at diagnosis. Mean hemoglobin, sodium, phosphor, calcium and serum albumin of patients were 10.60 mg/dl, 138.76 mEq/L, 5.15 mg/dl, 8.75 mg/dl and 3.66 g/dl, respectively.

###  Survival time of patients

Laplace regression results showed that the 10, 20, 30 and 40 percent of the investigated HD patients died in the 5.63, 13.43, 22.27 and 33.13 months after the diagnosis, respectively. The effect of prognostic factors on patient’s survival has been demonstrated in Table 1. Based on multivariable Laplace regression, females had 3.3 months’ higher survival (40^th^ percentile difference = 3.29 months, 95% CI: 0.69, 5.88). Urban residence patients had 2.65 months more survival compared with rural dwellers (40th percentile difference = 2.65 months, 95% CI: 0.50, 4.80). We observed a significantly higher survival in nonsmoker patients (40^th^ percentile difference = 5.34 months, 95% CI: 2.06, 8.61). Survival was shortened by more than 3 years in CRP positive patients (40^th^ percentile difference = 36.90 months, 95% CI: 32.37, 41.42). Patients with normal Albumin ≥3.5 g/dl) (40^th^ percentile difference =24.92, 95% CI: 18.04, 31.80) and normal Hemoglobin ≥11 g/dl) (40^th^ percentile difference = 18.65, 95% CI: 12.43, 24.86) had significantly higher survival (P<0.001).

**Table 1:** 40^th^ Percentile in survival and its difference according to levels of predictors, in HD patients in Hamadan province, 2007-2017

**Variables**	**No (%)**	**Time to death** **(months)**	**Unadjusted Percentile** **Difference (95% CI)**	***P *** **value**	**Adjusted** **percentile** **Difference (95% CI)** ^a^	***P*** ** value**
Gender						
Male	617 (54.03)	25.56	Ref.		Ref.	
Female	525 (45.97)	30.79	5.23 (-1.23,11.68)	0.120	3.29 (0.69, 5.88)	0.013
Location						
Urban	717 (62.78)	40.95	Ref.		Ref.	
Rural	425 (37.22)	35.3	5.65 (-0.52, 11.82)	0.070	2.65 (0.50, 4.80)	0.016
Education (yr)						
≤9	939 (82.22)	21.92	Ref.			
>9	203 (17.78)	31.89	9.97 (-0.06, 20.00)	0.050	-	-
Body mass index (kg/m^2^)						
<25	820 (71.80)	33.90	Ref.			
≥25	322 (28.20)	36.07	2.17 (-5.10, 9.44)	0.560	-	-
Smoking						
Yes	256 (22.42)	25.98	Ref.		Ref.	
No	886 (77.58)	34.97	8.99 (0.38, 17.61)	0.040	5.34 (2.06, 8.61)	0.001
Albumin (g/dl)						
<3.5	427 (39.28)	10.57	Ref.		Ref.	
≥3.5	660 (60.72)	35.49	24.92 (18.04, 31.80)	0.001	12.34 (9.02, 15.67)	0.001
Phosphor (mg/dl)						
<3.5	114 (10.22)	22.28	Ref.			
≥3.5	1002 (89.78)	27.93	5.65 (-4.90, 16.19)	0.290	-	-
Calcium						
<8.5	333 (29.73)	29.94	Ref.			
≥8.5	787 (70.27)	31.77	1.83 (-5.00, 8.68)	0.600	-	-
**Sodium**						
<135	214 (19.18)	34.12	Ref.			
≥135	902 (80.82)	33.52	0.60 (-6.80, 800)	0.880	-	-
C-reactive protein						
Positive	385 (34.65)	8.77	Ref.		Ref.	
Negative	726 (65.35)	45.67	36.90 (32.37, -41.42)	0.001	16.46 (12.68, 20.23)	0.001
Dialysis weekly (hour)						
<10	405 (35.46)	23.40	Ref.			
≥10	737 (64.54)	29.34	5.94 (-1.17, 31.00)	0.110	-	-
Hemoglobin (mg/dl)						
<11	588 (53.60)	16.63	Ref.		Ref.	
≥11	509 (46.40)	35.28	18.65 (12.43, 24.86)	0.001	22.42 (18.10, 26.74)	0.001
Urea reduction ratio						
<0.65	512 (49.04)	24.22	Ref.			
≥0.65	532 (50.96)	29.82	5.60 (-1.60, 12.76)	0.130	-	-

^a^ Adjusted for other variables in the model

###  The interaction between smoking and CRP status

**Figure 1:**
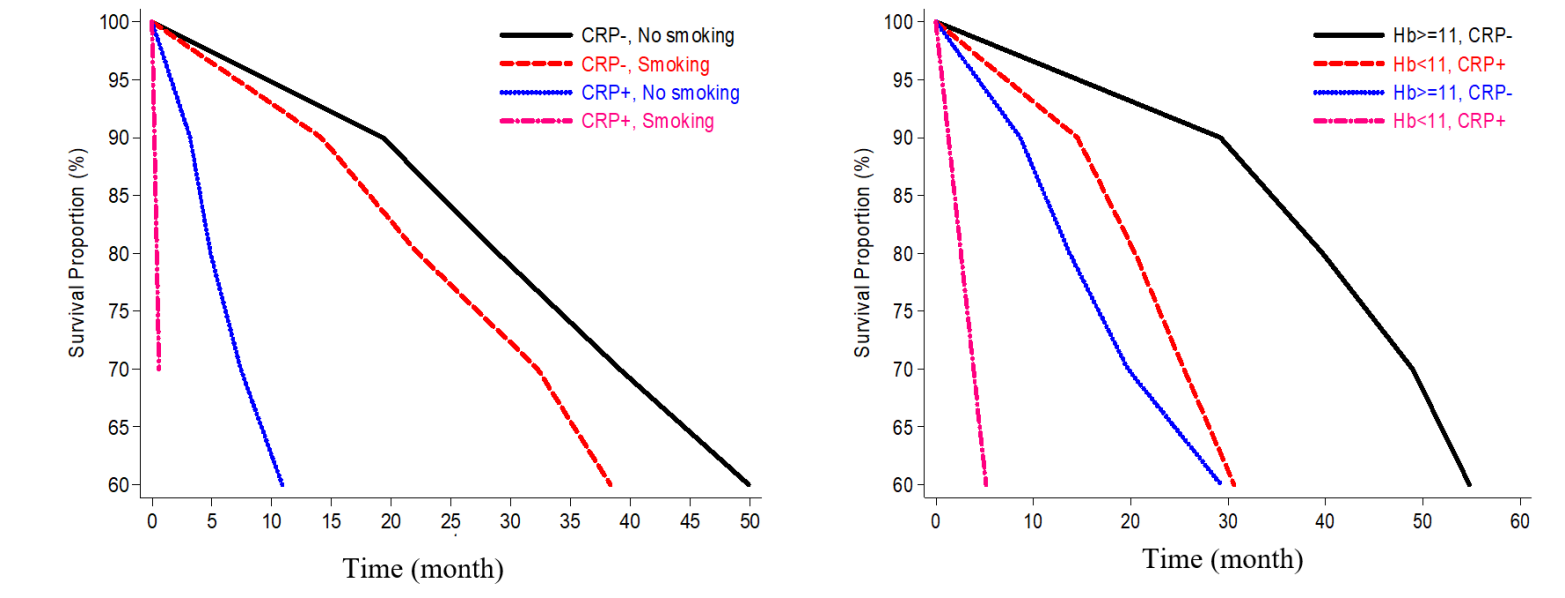
Survival curves (according Laplace regression approach) by levels of **a.** smoking (smoker, never smoker) and CRP status (positive, negative); **b.** hemoglobin (<11 mg/dl, ≥11 mg/dl) and CRP status (positive, negative)

The additive interaction between smoking (0 = smoker; 1 = never smoker) and CRP (0 = CRP positive; 1 = CRP negative) in predicting overall mortality were assessed. Predicted values of the 40^th^ survival percentile for each of the four subgroups formed, calculated by combining the obtained coefficients estimates, have been shown in Table 2 and Figure 1a. The Laplace regression model indicated superadditive interaction in predicting the death between being nonsmoker and CRP negative: 9.42 months’ excess in 40^th^ survival percentile for the effect of one predictor when the other predictor changes from 0 to 1. (β3 = 9.42 months, 95% CI: 3.35, 15.49 (*P*=0.002)).

###  The interaction between Hemoglobin level and CRP status

The interaction between Hemoglobin level (0 = Hb<11 mg/dl; 1 = Hb ≥11 mg/dl) and CRP status (0 = CRP positive; 1 = CRP negative) in predicting overall mortality in 40^th^ survival percentile of HD patients has been shown in Table 3 and Figure 1b.

**Table 2:** 40th Survival percentiles (months), by levels of Smoking and CRP status

**Variables**	**Smoker**	**Never smoker**	**Percentile** **difference**
CRP Positive	7.03	9.91	2.88
CRP Negative	37.15	49.45	12.30
Percentile difference	30.12	39.54	9.42

The estimate of the product term β_3_, suggested 5.26 additional months of 40^th^ survival percentile for the effect of one variable when the level of the other one varies from 0 to 1 (β_3_ = 5.26 months, 95% CI: -3.31, 14.81 (*P*=0.21)). This excess in survival indicates the presence of superadditive interaction in predicting mortality between being normal hemoglobin level and CRP negative.

**Table 3:** 40th Survival percentiles (months), by levels of Hemoglobin and CRP status

**Variables**	**CRP Positive**	**CRP Negative**	**Percentile difference**
Hb<11 mg/dl	7.40	29.80	22.40
Hb ≥11 mg/dl	26.40	54.07	27.67
Percentile difference	19.00	24.27	5.27

## Discussion

 Hemodialysis is a common treatment modality for ESRD patients in Iran. We investigated the predictors of survival in HD patients and explored the additive interaction between them in terms of survival. The findings of this study are useful as preliminary data for further studies in order to increase the quality care of HD patients. High CRP and low serum albumin and hemoglobin are independent predictors of mortality in HD patients. Being male, rural dweller and smoking were associated with a higher risk of mortality. There was an additive interaction between CRP and each of the variables serum hemoglobin and smoking on the (40th percentile of) survival.

In this regard, the low level of hemoglobin was associated with a higher risk of mortality in HD patients, which is consistent with our findings ^15,16^. In a study, three-year survival rate of patients with Hb <9 g/dL was significantly lower than that for patients with Hb levels 10 to 11 g/dL (74.1% vs. 89.3%) ^8^. The normal range of the hemoglobin level in HD patients is correlated with improving life quality ^17^, cardiac and brain function ^18,19^, decreasing hospitalization and treatment costs in these patients ^20^.

In the present study, in agreement with several conducted observational studies, we found that low level of serum albumin was associated with poor survival ^8,21,22^. Decreasing serum albumin levels with increasing time was associated with raising CVD related death ^23^. In dialysis patients, hypoalbuminemia is applied as indicator of malnutrition and has a strong effect on mortality ^24^.

Our findings revealed that the positive CRP was a strong predictor of death in HD patients. In consistence with our findings, several prospective studies have demonstrated CRP is an independent predictor for the future risk of death in these patients ^25,26^. In general, inflammation is associated with wasting, oxidative stress, insulin resistance, endothelial dysfunction, and infections ^17^. CRP can mediate processes involving in the development of atherosclerosis through plaque initiation, formation, and rupture, while it may not be merely a marker of inflammation ^27^.

Our results showed a significant association between HD patients’ smoking and their survival rate, which was inconsistent with the findings of another study, which showed there was no significant relationship between smoking and survival rate ^28^. A meta-analysis study reported a same results as our findings ^29^.

Smoking as a modifiable risk factor for kidney failure through some mechanisms like excessive generation of free radicals, promoting atherosclerosis in renal arteries, and intra-renal hemodynamic changes ^30,31^.

Additive interaction scale as a more relevant public health measure helps detect subgroups with the highest benefits from treatment ^10^. The findings of this study support the presence of the super additive interaction between CRP status and serum Hemoglobin also CRP status and smoking. This finding is important because HD patients with a high mortality risk can be identified through regular screening. More studies are required to determine that multiple pathophysiological pathways may underlie these interaction effects.

 Our study has suffered from some key limitations which had been inherited from existing data. Firstly, the absence of a Kt/V as an accurate reference method to estimate dialysis adequacy of patients. Second, due to its retrospective design, it was not possible to control data quality. Third, the addiction and smoking status of patients was based on their self-report and were prone to measurement bias. Fourth, the retrospective and observational nature of our analyses allowed the detection of associations, not causation. However, moderately large sample size, comprehensive clinical and laboratory evaluations and examining the additive interaction between the predictors of HD patient’s survival, can be considered as the strengths of our study.

## Conclusion

 High CRP and low serum albumin and hemoglobin are associated with the increased risk of death in HD patients, and male gender, rural dweller and smoking were significantly associated with a higher risk of mortality. The presence of super-additive interaction between CRP status and serum hemoglobin also CRP status and smoking, resulting in excess survival in HD patients. These findings can help screening programs to identify patients with a high mortality risk.

## Acknowledgements

 This study is a part of thesis in Ph.D. of Epidemiology at Tehran University of Medical Sciences (Research code: 9221128004). We would like to thank parson participating in this project and the health staff of the hemodialysis wards in hospitals of Hamadan Province for their kind cooperation.

## Conflict of interest

 The authors declare that there is no conflict of interest.

## Funding

 This study has been supported by Tehran University of Medical Sciences.

## Highlights

- CRP positive and low level of serum albumin and hemoglobin were associated with increasing death risk in hemodialysis patients.
- There is a super-additive interaction between CRP status with serum hemoglobin in excess survival of hemodialysis patients.
- There is a super-additive interaction between CRP status with smoking in excess survival of hemodialysis patients.
